# Comparison of five conjunctival cytology sampling methods in normal cat eyes

**DOI:** 10.14202/vetworld.2023.779-785

**Published:** 2023-04-15

**Authors:** Liga Kovalcuka, Liga Sarpio, Madara Nikolajenko

**Affiliations:** 1 Clinical Institute, Faculty of Veterinary Medicine, Latvia University of Life Sciences and Technologies, Jelgava, Latvia; 2 Vetclinic24, IVC Evidensia, Riga, Latvia

**Keywords:** cat, cytology, conjunctiva, sampling methods

## Abstract

**Background and Aim:**

Ophthalmological cytology is an easy, informative, rapid, and commonly-used low-cost diagnostic method, but sample collection and preparation are essential steps in obtaining qualitative material for cytological evaluation. This study aimed to evaluate cytological smear quality and animal discomfort after single or three serial conjunctival scrapings in normal cat eyes using five sampling methods.

**Materials and Methods:**

Five cytology methods (mini brush, cotton swab, soft brush, Kimura spatula, and cytobrush) were used in 50 eyes (10 with one scraping and 10 with three consecutive scrapings for a particular method) in complete 25 clinically and ophthalmologically healthy cats of different ages, sexes, and breeds. Ocular discomfort (1 = eyes open, 2 = partially open, and 3 = eyes squinted), average cell count (ten 10× fields), cell distribution (ten 100× fields: 0 = all cells are aggregated, 1 = <25% cells are evenly distributed, 2 = 25–50% cells are evenly distributed, and 3 = >50% cells are evenly distributed) and sample quality – aggregates (two cells and more), mucus, and artifacts (1+ = fair, 2+ = moderate, and 3+ = high amount) were evaluated.

**Results:**

The discomfort scores for the mini brush, cotton swab, soft brush, spatula, and cytobrush after a single and three scrapings were 1, 1, 1, 2, and 3, respectively. The average cell counts ± standard deviation after one and three scrapings were as follows: mini brush 11.15 ± 13.87 and 7.55 ± 12.7; cotton swab 7.17 ± 10.20 and 10.00 ± 16.44; soft brush 19.45 ± 22.22 and 8.55 ± 13.82; spatula 17.15 ± 32.94 and 13.85 ± 22.01; and cytobrush 13.35 ± 18.33 and 13.05 ± 19.29, respectively; the cell distributions were 3, 3, 3, 1, and 1 after single scraping and 3, 3, 2, 0, and 2 after three scrapings, respectively.

**Conclusion:**

The mini brush was the optimal method since it produced less discomfort, fewer artifacts, and the highest smear quality. Spatula smears were difficult to evaluate due to material thickness. The highest mucus and aggregate amounts were found in cytobrush, cotton swab, and soft brush samples. In this study, small number of samples per each sampling method is a major limitation.

## Introduction

Ophthalmological cytology is an easy, informative, rapid, and commonly-used low-cost diagnostic method that, in most cases, requires only basic knowledge of cytology. Cytology can provide the clinician with a causative diagnosis or help to guide further diagnostic workup [1]. Most frequently, conjunctival diseases in cats are caused by various infectious agents such as feline herpes virus-1, feline calicivirus, *Chlamydophila felis*, *Mycoplasma felis*, and various bacteria [2–4]. However, regardless of the cause, clinical signs are similar and include conjunctival edema, hyperemia, blepharospasms, and discharge [5, 6]. Specific diagnosis of the infectious disease can be attained using serology or polymerase chain reaction, but these tests are not performed in the clinic, are more time-consuming, and have a higher cost. In comparison, cytology is rapid, straightforward, and inexpensive, although cytologic sensitivity for feline herpesvirus-1 and chlamydophila is low [7–9]. Cytology is often used in the diagnosis of neoplasia and inflammation to support the analysis of immune-mediated diseases, eosinophilic diseases, allergies, and hypersensitivity, and it helps in guiding the next diagnostic steps as well as monitoring treatment efficacy [3].

Sample collection and preparation are essential steps in obtaining qualitative material for cytological evaluation. Cellularity and cell preservation are crucial, along with the skills of sample collection and interpretation. The conjunctiva is exposed to the environment; therefore, artifacts in cytology material can be present and mimic a pathogen or neoplastic cells [2, 6]. A cytology sample of excellent quality contains an evenly distributed monolayer of intact cells.

Cytological material can be obtained using various methods and tools. The most commonly used tools include cotton swabs, cytology brushes, Kimura spatulas, or the blunt ends of scalpel blades, and the last has the highest possibility of disrupting the normal cellular architecture [7, 10]. An impression smear does not greatly disrupt the cellular architecture, produces a good-quality sample, and causes minimal discomfort to cats. However, it has not been commonly used in daily clinical practice [1, 10, 11].

A few publications have compared fewer sampling methods [12, 13], but animal discomfort was not evaluated in those studies. Therefore, this study aimed to compare five different sampling techniques (mini brush, cotton swab, soft brush, spatula [Kimura platinum spatula], and cytobrush), evaluate the quality of the cytological smears after one and three scrapings of the conjunctiva, and for the 1^st^ time, to our knowledge, assess animal discomfort during sampling.

## Materials and Methods

### Ethical approval and informed consent

This study fully complied with the ethical standards and welfare of the cats involved. All examined animals were privately owned outpatients of the Latvia University of Life Sciences and Technologies, University Veterinary Clinic. The examination procedures performed during routine clinical and ophthalmology examinations did not exceed good veterinary practice principles and were not painful; therefore, an ethical permit for the protection of animal welfare was not required, although written informed client consent was obtained. To be included in the study, cats were required to be free of systemic and ocular disease and could not have undergone any administration of medication.

### Study period and location

The study was conducted from September 2020 to May 2022 at the Small Animal Clinic and Clinical Institute of the Faculty of Veterinary Medicine, University of Life Sciences and Technologies, Latvia.

### Animals and sampling

Twenty-five clinically and ophthalmologically healthy cats were included in the study, and 50 eye cytology samples were obtained. The demographics of the included cats were as follows: ten female and 15 male cats aged 4 months to 13 years, several different breeds [19 mixed breeds, oriental, burmese, bengal, maine coon, british shorthair, sphynx (one of each pure breed)], and different neuter status (19/25 were neutered or spayed).

All cats underwent ophthalmological examinations, including a slit lamp (Kowa SL15, Nagoya, Aichi, Japan), rebound tonometer (TonoVet®, Tiolat Ltd., Vantaa, Finland), direct ophthalmoscopy (Keeler Practitioner, Windsor, UK), monocular ophthalmoscopy with a PanOptic ophthalmoscope (Welch Alynn, Romford, UK), and tear production measured with standardized sterile Schirmer tear test (STT) strips (Eickemeyer, Tuttlingen, Germany). Genetic defects (posterior capsule cataract [2], posterior nuclear cataract [1]) were diagnosed in three cats; however, as the findings had no effect on the factors that were the focus of the study, those cats were not excluded from the study.

**Figure-1:**
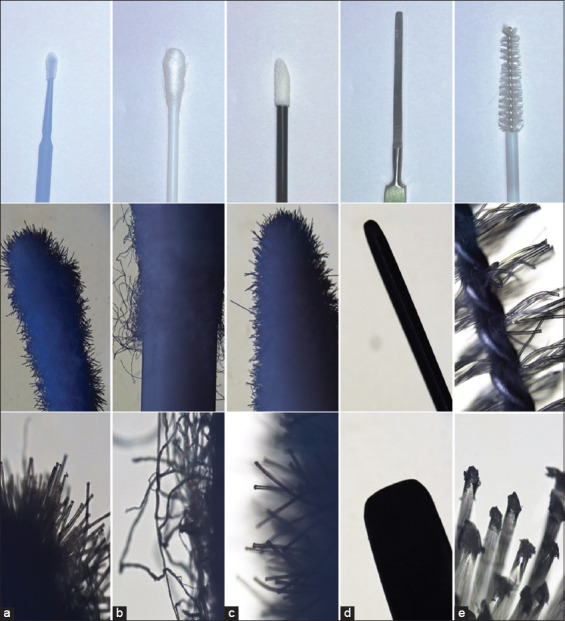
Cytological sampling instruments in native size. Line 1 (a) mini brush, (b) cotton swab, (c) soft brush, (d) spatula, (e) cytobrush. Line 2 (magnified 20×). Line 3 (magnified 100×).

Conjunctival cytology was performed at least 5 min after STT. Before obtaining the material, eye conjunctiva was cleaned of excess material and debris. Sample was obtained from the right eye first and then left eye with one of the following five randomly chosen sterile instruments: mini brush, cotton swab, soft brush, spatula (Kimura platinum spatula), and cytobrush (Figure-1). In total, ten samples were collected using each instrument. Five samples were obtained by gently scraping the conjunctiva once, and five samples were obtained by gently scraping 3 times (Table-1) [14]. Before procuring a sample, a topical proxymetacaine hydrochloride (5 mg/mL; Alcaine, Alcon-Couvreur, Belgium) anesthetic eye drop was applied for at least 30 s as recommended for cytology sampling [15]. The lower eyelid was pulled down to expose the inferior conjunctival fornix, and a brush was carefully pulled over the conjunctiva to collect the sample.

**Table-1:** Number of cytology samples per method.

Sample type	Mini brush	Cotton swab	Soft brush	Spatula	Cytobrush
1 scraping	5	5	5	5	5
3 scrapings	5	5	5	5	5
Complete sample amount	10	10	10	10	10

Collected material from the conjunctiva was gently transferred to a glass slide. The material from the mini brush, cotton swab, and cytobrush was gently rolled over the slide. The material from the spatula and soft brush was gently smeared on the slide. Each slide was marked, air-dried, and stained using the Romanowsky Diff Quick method [14, 15].

While obtaining a cytological sample, the discomfort was assessed according to Feline grimace scale action unit: the orbital tightening (1 = eyes opened, 2 = eyes partially opened, and 3 = or eyes squinted) [16].

Cytological samples were examined using an optical light microscope under 10× magnification to assess sample quality and cell distribution and with 100× magnification in oil immersion for cell count in the field of view. Analytical criteria, including cellularity (total and average cell count [intact epithelial cells, damaged epithelium], Goblet cells, and intact leukocytes [including differentiating of leukocytes in the total and average cell count] and artifacts in ten vision fields); distribution (0 = all cells are aggregated, 1 = <25% cells are evenly distributed, 2 = 25–50% cells are evenly distributed, and 3 = >50% cells are evenly distributed); and sample quality – aggregates (2 cells and more), mucus, and artifacts (where 1+ = fair amount, 2+ = moderate amount, and 3+ = high amount). The sample quality was determined by counting the “+” from each sample quality parameter together and calculating the mean values from the sum.

Cytological evaluations were performed masked to the type of cytology method and the number of scrapings.

### Statistical analysis

Statistical analyses were performed using statistical product and service solutions (the statistical package for the social sciences [SPSS], version 12.0.0, SPSS Inc., Chicago, IL, USA and Microsoft Office, Excel, version 2016, Microsoft Corp, Redmond, USA); p < 0.05 was considered statistically significant.

## Results

Fifty samples obtained with different tools from 25 cats were evaluated in this study. The degree of discomfort, average cell count, cell distribution, sample quality, and several artifacts were analyzed for each of the samples. The sample-quality analysis scores from the spatula scrapings were mostly invalid because most samples contained aggregates. Consequently, these samples were not considered appropriate material, and further, calculations were not performed.

### Discomfort

The discomfort scores were observed and calculated using mode. The scores for one and three scrapings for mini brush, cotton swab, soft brush, spatula, and cytobrush were 1, 1, 1, 2, and 3, respectively (Table-2). None of the animals showed continued eye redness or epiphora 20 min after sampling.

**Table-2:** Animal discomfort 1–3 (1=eyes opened, 2=eyes partially opened, 3=or eyes squinted) [14].

Cytology method	1 eyes opened (no discomfort)		2 eyes partially opened (minimal discomfort)		3 eyes squinted (shows resistance, shows pain)	
		
	1×	3×	1×	3×	1×	3×
Mini brush	•	•				
Cotton swab	•	•				
Soft brush	•	•				
Spatula			•	•		
Cytobrush					•	•

1×=Single scraping, 3×=3 times scraping

### Sample cellularity

**Figure-2:**
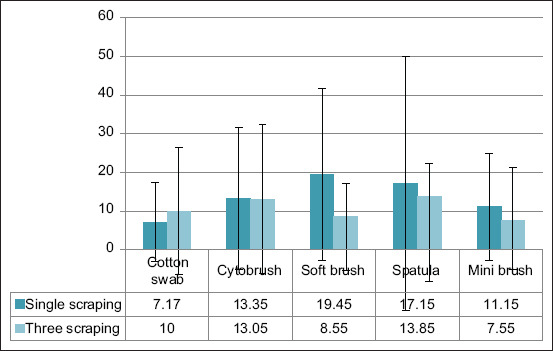
Cell average count with a standard deviation.

The average cell count ± standard deviation after 1 scraping for mini brush, cotton swab, soft brush, spatula, and cytobrush were 11.15 ± 13.87, 7.17 ± 10.20, 19.45 ± 22.22, 17.15 ± 32.94, and 13.35 ± 18.33, respectively, and those for three scrapings were 7.55 ± 12.7, 10.00 ± 16.44, 8.55 ± 13.82, 13.85 ± 22.01, and 13.05 ± 19.29, respectively (Figure-2). These results demonstrated that the soft brush and cytobrush produced the highest cell counts.

### Cell distribution

Distribution was assessed, and each sample was categorized. The distribution of the cells was calculated using the mode for each technique. These values after 1 scraping for mini brush, cotton, soft brush, spatula, and cytobrush were 3, 3, 3, 1, and 1, respectively, and those after three scrapings were 3, 3, 2, 0, and 2, respectively. The poorest cell distribution was observed with cytobrush, and the spatula samples showed aggregation (Table-3).

**Table-3:** Cell distribution 0–3; 0=all cells are aggregated, 1 = <25% cells are evenly distributed, 2=25–50% cells are evenly distributed, 3 = >50% cells are evenly distributed.

Cytology method	0-all cells are aggregated		1- <25% of cells are evenly distributed		2-25–50% cells are evenly distributed		3- >50% of cells are evenly distributed	
			
	1×	3×	1×	3×	1×	3×	1×	3×
Mini brush							•	•
Cotton swab							•	•
Soft brush						•	•	
Spatula		•	•					
Cytobrush			•			•		

1×=Single scraping, 3×=3 times scrapings

### Sample quality

**Figure-3:**
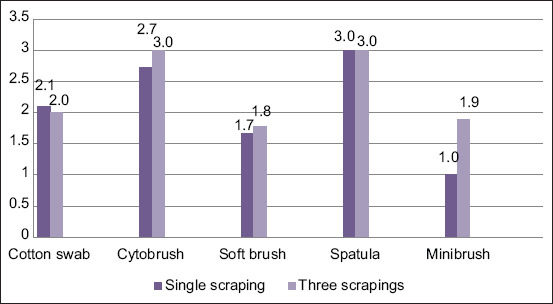
Sample quality. Aggregates, mucus, and artifacts where 1+ = fair amount, 2+ = moderate amount, and 3+ = high amount.

The results for mini brush, cotton swab, soft brush, spatula, and cytobrush after 1 scraping were 1, 2.1, 1.7, 3, and 2.7, respectively, and those for three scrapings were 1.9, 2, 1.8, 3, and 3, respectively (Figure-3). Sample quality was poorest in samples taken with a cotton swab or cytobrush.

## Discussion

To the best of our knowledge, only a few existing studies have compared different cytological sampling methods, and none have evaluated the discomfort caused by conjunctival sampling. Therefore, in this study, we compared five different sampling techniques with respect to smear quality and animal discomfort during sampling. Three of these techniques were commonly used (spatula, cytobrush, and cotton swab) in the literature [7, 17]. In contrast, the mini brush has been reported and used only recently [13], and the soft brush, to our knowledge, has not been used for this purpose to date.

The sampling method is a crucial step for obtaining a high-quality sample. However, it is imperative [17] that the instruments used for sampling are minimally invasive and comfortable for the animal [11, 18]. In human medicine, the Ocular Surface Disease Index was developed by the Outcomes Research Group (Allergan Inc., Irvine, CA, USA) to assess the discomfort of the eye surface [19]. Similarly, one report exists on the horse ophthalmic pain scale; however, in cats for general acute pain assessment, Feline grimace scale [16] is used, up to date, no pain scale has been developed for feline ocular discomfort.

### Mini brush

**Figure-4:**
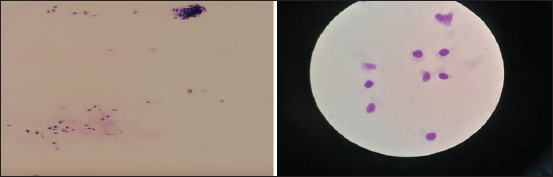
Mini brush cytology sample-to the left 20× magnification, to the right 100× magnification.

The mini brush was introduced as a new sampling method. It caused no discomfort after a single- and 3-time scrapings. No other assessment of mini brush discomfort associated with sampling exists in the literature. Moreover, we agree with the recent opinion of Ripolles-Garcia *et al*. [13] that the mini brush can be used to access small, limited, and specific regions and, at the same time, minimize the potential risks for iatrogenic damage to the conjunctiva or cornea. We agree that the mini brush should be used in deep ulcers or keratomalacia to achieve non-invasive and precise sampling from a small, local area. In our hands, it provided high-quality cells and distribution (Figure-4). Most of the cells were present in a monolayer, with very few artifacts or mucus, enabling straightforward examinations. The only disadvantage that we observed was the poor cellularity of the sample, which could be due to the sample collection technique since it differed from that described in other studies [12, 20]. Nevertheless, the results were very similar, and we believe that this is due to the small surface area of the brush structure (Figure-1).

### Cotton swab

**Figure-5:**
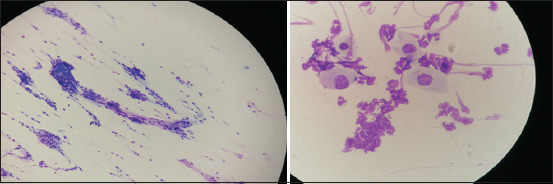
Cotton swab cytology sample-to the left 20× magnification, to the right 100× magnification.

No discomfort was evident after a cotton swab was used for scraping once or 3 times. In addition, Athanasiou *et al*. [20] have mentioned that topical anesthesia is rarely required for the cotton swab method because it is well tolerated and has been recommended for use in deep corneal ulcers or keratomalacia. Cotton swabs are readily available in the clinic; however, those most in use are not usually of the highest quality. In this study, dry cotton swabs produced a large amount of compact mucus and aggregates with artifacts (striped material, Figure-5), and a relatively small number of cells, as described previously [12, 21]. This effect could be due to the cotton fibers (Figure-1), which also persisted on the slides as foreign bodies. Before obtaining the material, it is advised to clean the eye conjunctiva of excess material and debris [20] to reduce the artifacts. However, in this study, the artifacts were still significantly present even after cleaning. The cotton swab mode of action is to absorb fluids rather than to attract cells. Therefore, cotton swabs are preferred for microbiology over cytology [21].

### Soft brush

**Figure-6:**
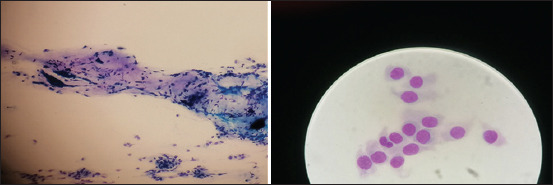
Soft brush cytology sample-to the left 20× magnification, to the right 100× magnification.

The other new method uses a soft brush made from upholstery material that is typically used in cosmetology (Figure-1). In our study, this brush proved to be quite similar to a cotton swab in that it caused no discomfort after a single scraping and minimal resistance after three scrapings, but it produced a higher cell count than cotton swabs (Figure-6). The sample quality was also higher than that obtained with cotton swabs, which could be due to the smoother surface of the soft brush versus the uneven surface of the cotton swab due to the cotton fibers (Figure-1). The samples obtained with the soft brush were cell-rich and compact (Figure-6) but could still provide enough cells for evaluation. However, compared to the mini brush, the soft brush was of inferior quality and resulted in poor cellular distribution and higher animal discomfort. This, we concluded that a soft brush should not be the preferred sampling tool.

### Spatula (Kimura platinum spatula)

**Figure-7:**
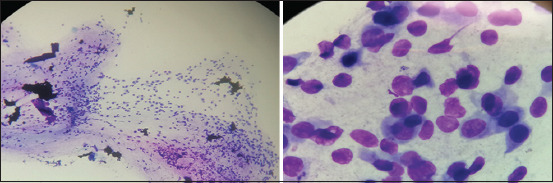
Spatula cytology sample-to the left 20× magnification, to the right 100× magnification.

Minimal discomfort was observed with the spatula after a single and three scrapings. Caution should be used to avoid rupturing the globe during the scraping of the tissue. The round straight end of the spatula must be used to scrape with quick light movements in the same direction against the conjunctiva, thereby avoiding traumatization and preserving cellular morphology [20]. The spatula is considered the gold standard method in the literature [1, 20]. Cytological samples usually contain high numbers of cells, and aggregates can be present, making evaluation challenging [1, 12]. In this study, samples obtained by spatula were difficult or impossible to examine due to the number of aggregates making up nearly 95% of all material (Figure-7). Consequently, most of the calculations for this method were not valid due to the aggregate problem, which was evident in the high standard deviations observed (Figure-3). Moreover, it was impossible to examine the material and, therefore, to diagnose any pathology using this method. Our finding of such a poor result with this instrument is not consistent with results from other studies showing successful application of the spatula [12, 20, 22]. The different results might be owing to technique or another human factor.

### Cytobrush

**Figure-8:**
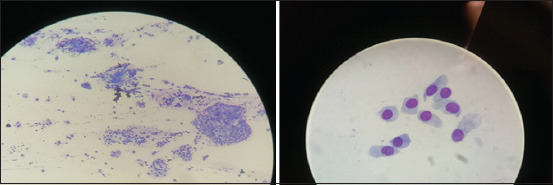
Cytobrush cytology sample-to the left 20× magnification, to the right 100× magnification.

The most animal discomfort was observed after a single and three scrapings using the cytobrush. The discomfort was communicated by eye closing, demonstrating resistance to the procedure and causing a lower recommendation for this method. We presume that this finding is due to the structure of the cytobrush that is in contrast to the soft, short nylon bristles of the mini brush. The cytobrush also has nylon brushes, but they are a lot larger, thicker, and more abrasive (Figure-1). The cytobrush is used worldwide for cytological sampling from any type of mucous membrane, including the conjunctiva, and has been described as providing good cellular distribution with a monolayer, but it produces a lower cell count than other methods [12, 20]. In this study, we observed the opposite and detected a high cell count (Figure-8), many aggregates, and abundant mucus and artifacts after using the cytobrush. Notwithstanding, we also observed many high-quality cells that were diagnostic, but when compared to the amount of artifacts, those cells were in the minority.

## Conclusion

Overall, cytology is a valid diagnostic tool in ophthalmology. It can provide rapid and beneficial answers and guide further diagnostics. In our study, the spatula was the only method/tool characterized as a non-recommended method proving low-quality samples. The other four methods were adequate; however, in our opinion, the use of the mini brush was the best method. This method caused no or minimal discomfort and produced a high-quality sample with a monolayer of cells. We suggest the use of a new type of mini brush could be extrapolated to use in corneal ulcers to obtain cells from a more precise location. In this study, a small number of samples per sampling method was a limitation.

## Authors’ Contributions

LK: Conceptualized the aim of the study, designed, planned, and supervised the study and corrected the manuscript. LS, MN, and LK: Conceived the work and performed all animal examinations and tests. LS: Analyzed the data, prepared the graphs, figures, and tables, and drafted the manuscript. All authors have read, reviewed, and approved the final manuscript.
